# Effects of add-on transcranial direct current stimulation on pain in Korean patients with fibromyalgia

**DOI:** 10.1038/s41598-020-69131-7

**Published:** 2020-07-21

**Authors:** Ji-Hyoun Kang, Sung-Eun Choi, Dong-Jin Park, Haimuzi Xu, Jung-Kil Lee, Shin-Seok Lee

**Affiliations:** 1 Division of Rheumatology, Department of Internal Medicine, Chonnam National University Medical School and Hospital, 42 Jebong-ro, Dong-gu, Gwangju, 61469 Republic of Korea; 2 Department of Neurosurgery, Chonnam National University Medical School and Hospital, Gwangju, Republic of Korea

**Keywords:** Skeletal muscle, Fibromyalgia

## Abstract

Despite promising preliminary results of transcranial direct current stimulation (tDCS) treatment in patients with fibromyalgia (FM), several issues need to be addressed, including its limited efficacy, low response rate, and poor tolerability. We investigated the efficacy and safety of tDCS as an add-on treatment for chronic pain in Korean patients with FM. This study enrolled 46 patients who were refractory to pain medications from May 2016 to February 2017. A conventional tDCS device was used to supply 2 mA of current for 20 min on five consecutive days. The primary end-point was a change in visual analogue scale (VAS) pain score at the end of treatment; secondary end-points included changes in Fibromyalgia Impact Questionnaire (FIQ), Brief Pain Inventory (BPI), Brief Fatigue Inventory (BFI), Beck Depression Inventory (BDI), State-Trait Anxiety Inventory (STAI), and Medical Outcomes Study Sleep Scale (MOS-SS) scores. After tDCS, 46 patients showed clinical improvements in VAS pain scores on days 6, 13, and 36 compared with day 0 (p < 0.001). Improvement in FIQ was seen on day 13. The BDI decreased significantly on days 6 and 36, and BFI improved significantly on days 6 and 13. There were no significant improvements in STAI-I, STAI-II, and MOS-SS scores after tDCS. No serious adverse events were observed. Our results suggest that tDCS can result in significant pain relief in FM patients and may be an effective add-on treatment.

## Introduction

Fibromyalgia (FM) is a chronic pain disorder characterized by widespread pain and associated symptoms such as emotional distress, fatigue, and sleep disturbances. Although the pathophysiology of FM remains unclear, it is believed to be associated with changes in pain and sensory processing in the central nervous system, especially nociceptive pathways. These changes may be caused by maladaptive plasticity in pain-associated neural circuits^1–4^. In addition, dysregulated neurotransmitters in FM patients can result in exaggerated central sensitization to pain^5^.

Based on the mechanism described above, recent studies have shown that noninvasive brain stimulation techniques may be beneficial for chronic pain syndromes, including FM. Among the brain stimulation techniques, transcranial direct current stimulation (tDCS) is a novel treatment in FM patients with chronic pain. A previous randomized controlled trial by Fregni et al. reported the beneficial effect of tDCS treatment^6^. Other studies^7–9^ also showed that tDCS could induce pain reduction and maintain an improved pain status in FM patients. However, a Cochrane review^10^ noted that tDCS leads to small, short-term reductions in pain, and these effects are not likely important clinically and were effective in only a small number of FM participants. The number of published clinical trials was small and most of the trials had small sample sizes and short follow-up durations^10–12^. In addition, previous trials are biased by a lack of adequate blinding. Taken together, the evidence in support of tDCS remains insufficient. In addition to its efficacy toward pain intensity, little is known about the effect of tDCS on associated symptoms, such as fatigue and depression. No study has explored the booster effect of tDCS in FM patients who initially responded to tDCS to maintain the treatment effects. Because the effect sizes for pharmacological treatment are generally small, it is necessary to investigate whether tDCS is effective in patients who are refractory to pain medication. Thus, we explored the efficacy, tolerability, and safety of tDCS treatment as an add-on treatment for chronic widespread pain in a large number of Korean patients with FM.

## Methods

### Population and study design

We enrolled FM patients who were diagnosed and treated in the Department of Rheumatology at Chonnam National University Hospital in Korea from July 2016 to February 2017. All patients satisfied the 2010 American College of Rheumatology (ACR) criteria of FM^13^. The inclusion criteria were as follows: (1) Pain refractory to common analgesics and medications, (2) presenting pain Visual Analogue Scale (VAS) ≥ 5, (3) revised Fibromyalgia Impact Questionnaire (FIQ) ≥ 50, (4) underwent brain magnetic resonance imaging (MRI) within 1 year, and (5) signed informed consents after appropriate explanation from a practicing physician. Exclusion criteria were as follows: (1) patients who had severe psychological disorders, (2) brain disease such as epilepsy, stroke, traumatic injury, or tumor, (3) history of brain surgery, (4) intracranial implant or appendages, (5) developmental disorder, (6) drug abuse, and (7) pregnancy. A total of 46 participants were enrolled in this study. Patients were medicated with the same dosage of their prescribed medication at the time of enrollment and during the study period. All enrolled patients provided written informed consent at the time of enrollment in this study. This study was approved by the Institutional Review Board/Ethics Committee of Chonnam National University Hospital (CNUH-2016-124, date of approval 08/06/2016) and was registered with the Korean National Clinical Research Information Service (CRIS-KCT0002035, date of registration 02/09/2016). This study was conducted in accordance with the Declaration of Helsinki and the Good Clinical Practice guidelines.

### Study design

This study lasted 6 weeks including a 1-week screening phase, a 1-week tDCS treatment phase, and a 4-week follow-up observation phase in each patient. To investigate the efficacy of tDCS treatment, we assessed pain VAS scores and associated symptoms using several parameters. We assessed the pain VAS score on day 0 (pre-treatment, baseline), days 1–5 (during treatment), day 6 (1 day after treatment), day 13 (treatment after 1 week), and day 36 (treatment after 1 month). Other parameters, including revised FIQ, brief pain inventory (BPI), Beck Depression Inventory (BDI), brief fatigue inventory (BFI), state-trait anxiety inventory (STAI), and the medical outcomes study sleep scale (MOS-SS), were assessed on days 0 (pre-treatment, baseline), 6, 13, and 36 (post-treatment).

All patients were scheduled to receive tDCS treatment in five daily sessions (Monday to Friday, days 1–5) for five consecutive days. Conventional tDCS devices were connected to multichannel stimulation adaptors and 2-mA intensities of current were applied across the anode and cathode for 20 min. The anode electrode was placed over the M1 cortex (using the 10/20 system of electrode placement) and the cathode electrode was placed over the contralateral M1 cortex area. After applying gel on the sponges covering the anode and cathode, the physician positioned electrodes on the correct areas. The physician then placed a net-like hat on the patient’s head for immobilization.

### Outcome measures

The primary efficacy outcome was the change in pain VAS score from baseline (day 0) to treatment after 1 week (day 13). The pain VAS consists of a scale ranging from 0 (complete absence of pain) to 10 (corresponding to worst imaginable pain).

Secondary efficacy outcomes included revised FIQ, BPI, BDI, BFI, STAI, and MOS-SS: We evaluated changes in parameters from baseline (day 0) to 1 day after treatment (day 6), 1 week after treatment (day 13), and 1 month after treatment (day 36).

The quality of life and other domains of FM were measured using the Korean version of the revised FIQ^14^. The revised FIQ has 21 questions based on an 11-point numeric rating scale from 0 to 10, with 10 being worst. All questions were framed in the context of the past 7 days. The revised FIQ was divided into three linked sets of domains including function (9 questions), overall impact (2 questions), and symptoms (10 questions). The summed score for function (range 0–90) was divided by 3, the summed score for overall impact (0–20) was not changed, and the summed score for symptoms (0–100) was divided by 2. The total score is the sum of the three modified domain scores.

To evaluate psychiatric symptoms, we measured depression using the Korean version of BDI^15^. The BDI consisted of 21 multiple-choice questions; each item was rated on a four-point intensity scale and scores were added to give a total range from 0 to 63; higher scores represent more severe depression. To evaluate the presence and severity of anxiety, we used the Korean version of STAI^16^. This instrument, which consists of the STAI-I (anxiety concerning a specific event) and STAI-II (anxiety as a stable personality characteristics), has 40 items (20 items each in STAI-I and STAI-II).

The BPI is an instrument for evaluating pain that can measure the intensity of pain and the interference of pain with the patient’s life. Since pain can be variable throughout the day, the BPI asks patients to rate their pain intensity at the time of responding to the questionnaire (now) and at its worst, lowest, and average levels over the previous week. The rating can also be made for the last 24 h depending on the study design. The BPI also asks patients to rate how their pain interferes with their general activity, mood, walking, work, sleep, relationships, and enjoyment of life. We used the validated Korean version of the BPI^17^.

We measured fatigue severity using the Korean version of BFI^18^. The BFI consists of nine items on a single page. Fatigue and its interference are measured on numeric scales from 0 to 10. There are three items asking subjects to describe their fatigue now, at its usual level, and at its worst level during the previous 24 h using the measures “no fatigue” and “fatigue as bad as you can imagine.” The following six items describe how fatigue has interfered with aspects of their life during the previous 24 h. These items include general activity, mood, walking ability, normal work, relationships with others, and enjoyment of life. These interference scales range from 0 to 10. The global score for the BFI is calculated as the mean value of these nine items.

Sleep quality was measured using the MOS-SS^19^. The MOS-SS includes 12 items categorized into six dimensions assessing sleep disturbance, sleep adequacy, somnolence, quantity of sleep/optimal sleep, snoring, and awakening short of breath or with a headache.

### Safety

Safety outcomes were evaluated based on the presence and incidence of adverse events during the study. We monitored adverse events by interviewing patients after each session of tDCS and during the follow-up period using a check-list including dizziness, headache, sleep disturbance, skin reactions, etc.

### Statistical analysis

Statistical analyses were performed using SPSS for Windows software package (ver. 20; SPSS Inc., Chicago, IL, USA). To evaluate the effects of tDCS, paired t-tests were used to compare mean changes from baseline with various time points after treatment. The comparisons were performed using Bonferroni adjustments. A *p* value < 0.05 was considered significant.

## Results

A total of 46 patients with FM were enrolled in the present study and their baseline demographic and clinical characteristics were described in Table 1. The mean age of enrolled patients was 47.4 ± 10.0 years and 95.7% of patients were female. The mean disease duration was 31.5 ± 24.5 months. The baseline pain VAS score was 8.78 ± 1.15 and the FIQ score was 79.7 ± 14.9. All the patients finished five sessions of tDCS for five consecutive days.

**Table 1:** Baseline characteristics of 46 patients with fibromyalgia.

Characteristics	
Number	46
Age, years	47.4 ± 10.0
Gender (female)	44/46 (95.7%)
Disease duration, months	31.5 ± 24.5
Pain VAS	8.78 ± 1.15
FIQ	79.7 ± 14.9
BPI	96.4 ± 15.3
BFI	74.9 ± 11.6
BDI	33.8 ± 10.1
STAI-I	24.6 ± 9.3
STAI-II	28.4 ± 8.0
MOS-SS	39.0 ± 5.9

Unless otherwise specified, data are shown as means ± standard deviations.

*VAS* Visual Analogue Scale, *FIQ* Fibromyalgia Impact Questionnaire, *BPI* Brief Pain Inventory, *BFI* Brief Fatigue Inventory, *BDI* Beck Depression Inventory, *STAI* State-TrAIT ANXIETY INVEntory, *MOS-SS* Medical Outcomes Study Sleep Scale.

The primary endpoint of our study was VAS score on day 13 (treatment after 1 week). In the present study, the pain VAS score decreased significantly on day 6 (mean change from baseline: − 2.239; 95% confidence interval [CI] − 2.646 to − 1.833; *p* < 0.001), which was maintained on day 13 (treatment after 1 week) (− 2.891; 95% CI − 3.373 to − 2.409; *p* < 0.001) and day 36 (treatment after 1 month) (− 1.391; 95% CI − 1.959 to − 0.823; *p* < 0.001). In addition, there was significant reduction on day 13 (− 8.134; 95% CI − 12.65 to − 3.615; *p* = 0.009) compared with day 0 in FIQ. However, these decreases were not maintained at day 36.

After tDCS, BPI showed a significant improvement on day 6 (− 8.848; 95% CI − 13.95 to − 3.746; *p* = 0.009), which was also observed on day 13 (− 13.35; 95% CI − 18.79 to − 7.907; *p* < 0.001) and day 36 (− 9.253; 95% CI − 15.29 to − 3.216; *p* = 0.027). BFI decreased on day 6 (− 10.15; 95% CI − 14.94 to − 5.366; *p* < 0.001) compared with day 0. The improvement in these indices was observed on day 13. BDI also decreased significantly on day 6 (− 4.609; 95% CI − 7.915 to − 1.303; *p* = 0.006), which was maintained until day 36 (− 7.652; 95% CI − 10.67 to − 4.634; *p* < 0.001). However, there was no significant improvement in STAI-I, STAI-II, and MOS-SS after tDCS on days 6, 13, and 36 (Table 2).

**Table 2:** Follow up of outcome measures from baseline to each time points.

	Mean change from baseline (95% CI)	*p*-value	*p*-value adjusted by Bonferroni method
**Pain VAS score**			
Day 6	− 2.239 (− 2.646 to − 1.833)	< 0.001	< 0.001
Day 13	− 2.891 (− 3.373 to − 2.409)	< 0.001	< 0.001
Day 36	− 1.391 (− 1.959 to − 0.823)	< 0.001	< 0.001
**FIQ**			
Day 6	− 2.803 (− 5.993 to 0.387)	0.252	1.000
Day 13	− 8.134 (− 12.65 to − 3.615)	0.003	0.009
Day 36	− 2.437 (− 7.199 to 2.325)	0.308	1.000
**BPI**			
Day 6	− 8.848 (− 13.95 to − 3.746)	0.003	0.009
Day 13	− 13.35 (− 18.79 to − 7.907)	< 0.001	< 0.001
Day 36	− 9.253 (− 15.29 to − 3.216)	0.009	0.027
**BDI**			
Day 6	− 4.609 (− 7.915 to − 1.303)	0.002	0.006
Day 13	− 5.913 (− 10.15 to − 1.675)	0.021	0.063
Day 36	− 7.652 (− 10.67 to − 4.634)	< 0.001	< 0.001
**BFI**			
Day 6	− 10.15 (− 14.94 to − 5.366)	< 0.001	< 0.001
Day 13	− 9.565 (− 13.95 to − 5.179)	< 0.001	< 0.001
Day 36	− 4.804 (− 9.057 to − 0.552)	0.084	0.252
**STAI-I**			
Day 6	1.565 (− 0.251 to 3.382)	0.267	1.000
Day 13	− 1.239 (− 3.570 to 1.092)	0.870	1.000
Day 36	− 2.348 (− 5.132 to 0.436)	0.288	1.000
**STAI-II**			
Day 6	0.391 (− 1.598 to 2.380)	0.694	1.000
Day 13	− 0.761 (− 3.068 to 1.546)	0.510	1.000
Day 36	− 0.065 (− 2.363 to 2.233)	0.955	1.000
**MOS-SS**			
Day 6	0.239 (− 2.002 to 2.481)	0.831	1.000
Day 13	0.565 (− 1.532 to 2.663)	0.590	1.000
Day 36	− 0.869 (− 2.982 to 1.243)	0.411	1.000

*VAS* Visual Analog Scale, *FIQ* Fibromyalgia Impact Questionnaire, *BPI* Brief Pain Inventory, *BDI* Beck Depression Inventory, *BFI* Brief Fatigue Inventory, *STAI* State-Trait Anxiety Inventory, *MOS-SS* Medical Outcomes Study-Sleep Scale.

We next divided patients into groups based on the median pain VAS score, and the group with VAS scores greater than 8 showed a larger decrease in pain VAS scores on day 6 (− 2.74 ± 1.40 vs. − 1.53 ± 0.96; *p* = 0.003) and day 13 (− 3.48 ± 1.53 vs. − 2.05 ± 1.39; *p* = 0.006) compared with patients with pain VAS scores less than 8. However, there was no significant difference in pain VAS score between the two groups on day 36. In addition, patients with pain VAS scores greater than 8 showed a greater decrease in BFI on day 6 (− 15.6 ± 17.5 vs. − 2.47 ± 15.4; *p* = 0.009) compared with those having pain VAS scores less than 8. However, there was no significant difference in FIQ, BPI, BDI, STAI-I, STAI-II, and MOS-SS between the two groups (Table 3).

**Table 3:** Comparison of outcomes between the two groups according to the pain VAS score.

	Pain ≤ 8 (N = 19)		Pain > 8 (N = 27)	p-value	*p*-value adjusted by Bonferroni method
**Pain VAS score**					
Day 6	− 1.53 ± 0.96		− 2.74 ± 1.40	0.001	0.003
Day 13	− 2.05 ± 1.39		− 3.48 ± 1.53	0.002	0.006
Day 36	− 0.79 ± 1.51		− 1.82 ± 2.08	0.059	0.177
**FIQ**					
Day 6	− 0.60 ± 10.51		− 4.53 ± 10.80	0.246	0.738
Day 13	− 5.98 ± 13.73		− 9.65 ± 16.32	0.414	1.000
Day 36	0.41 ± 12.10		− 4.44 ± 18.31	0.284	0.852
**BPI**					
Day 6	− 7.16 ± 12.92		− 10.01 ± 19.81	0.554	1.000
Day 13	− 16.39 ± 21.31		− 11.22 ± 15.90	0.380	1.000
Day 36	− 8.62 ± 13.53		− 9.69 ± 24.32	0.081	0.243
**BDI**					
Day 6	− 4.74 ± 9.62		− 4.52 ± 12.30	0.946	1.000
Day 13	− 2.21 ± 18.72		− 8.52 ± 9.70	0.189	0.567
Day 36	− 8.32 ± 11.52		− 7.19 ± 9.34	0.725	1.000
**BFI**					
Day 6	− 2.47 ± 15.43		− 15.61 ± 17.50	0.003	0.009
Day 13	− 8.26 ± 13.52		− 10.52 ± 15.82	0.612	1.000
Day 36	− 0.68 ± 10.71		− 7.70 ± 15.91	0.081	0.243
**STAI-I**					
Day 6	1.37 ± 2.95		1.70 ± 7.66	0.837	1.000
Day 13	− 0.74 ± 5.15		− 1.59 ± 9.38	0.694	1.000
Day 36	− 3.11 ± 6.25		− 1.82 ± 11.10	0.620	1.000
**STAI-II**					
Day 6	− 0.42 ± 6.31		0.96 ± 7.02	0.488	1.000
Day 13	− 1.21 ± 6.39		− 0.44 ± 8.71	0.733	1.000
Day 36	− 1.37 ± 5.94		0.85 ± 8.78	0.312	0.936
**MOSS**					
Day 6	− 2.26 ± 7.13		2.00 ± 7.46	0.057	0.171
Day 13	− 0.95 ± 7.99		1.63 ± 6.27	0.249	0.747
Day 36	− 2.68 ± 7.31		0.41 ± 6.82	0.155	0.465

Values are shown as the mean ± standard deviation.

*VAS* Visual Analog Scale, *FIQ* Fibromyalgia Impact Questionnaire, *BPI* Brief Pain Inventory, *BDI* Beck Depression Inventory, *BFI* Brief Fatigue Inventory, *STAI* State-Trait Anxiety Inventory, *MOS-SS* Medical Outcomes Study-Sleep Scale.

**Figure 1:**
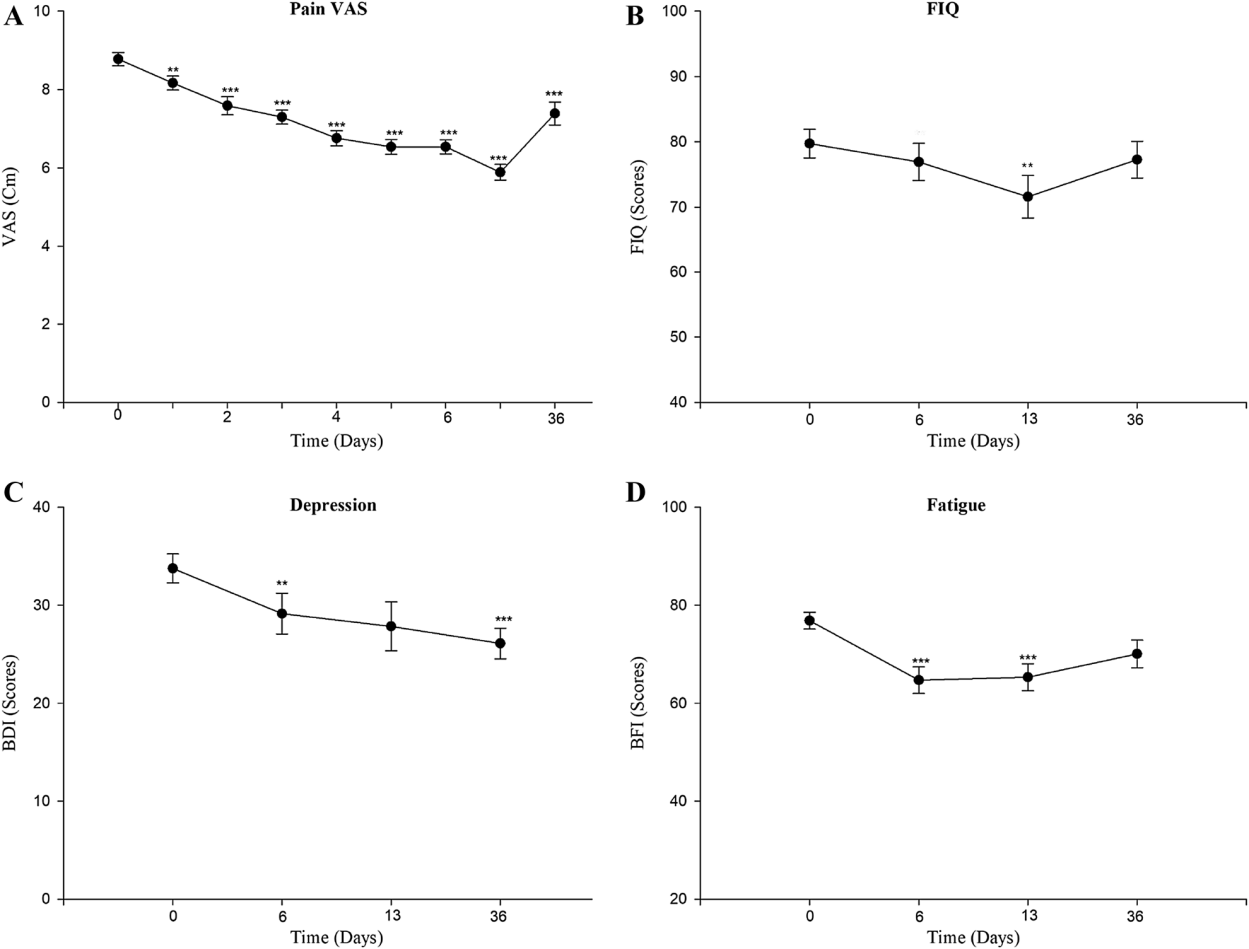
(**A**) Pattern of pain VAS scores by time in total patients. (pre-treatment, on treatment days 1–5, post-treatment 1-week, 1-month). (**B**) Pattern of FIQ scores by time in total patients. (**C**) Pattern of BDI scores by time in total patients. (**D**) Pattern of BFI scores by time in total patients. Values are shown as the mean ± standard error of the mean. *p < 0.05, **p < 0.01, ***p < 0.001. *VAS* Visual Analog Scale, *FIQ* Fibromyalgia Impact Questionnaire, *BDI* Beck Depression Inventory, *BFI* Brief Fatigue Inventory.

The slope for pain VAS scores in all patients with tDCS showed a significant decrease until day 36. From days 1 through 5, pain VAS scores decreased significantly compared to day 0 (Fig. 1A). The slope of FIQ decreased on day 13 (Fig. 1B). Additionally, the slope of BDI showed a significant reduction after tDCS treatment until day 36 (Fig. 1C) and the slope of BFI decreased on day 6 and day 13 (Fig. 1D).

Eight patients developed adverse events such as mild dizziness (5 patients), light headache (3 patients), and transient sleep disturbance (2 patients). However, there was no patient dropout or serious adverse event to prevent tDCS.

## Discussion

We observed a significant improvement in pain, quality of life, and fatigue in patients with FM after five daily sessions of tDCS, and the adverse effects of tDCS were minor and well tolerated.

This study showed that pain VAS scores improved significantly after tDCS treatment. Preliminary results of early studies^6–8^ exploring tDCS found significant pain reduction in FM patients. Those studies reported that the active tDCS group had a higher response rate and significant pain decrease compared with the sham group. Several issues were identified based on a recent meta-analysis and systematic review of tDCS^10–12^. Previous results have shown heterogeneity in the efficacy of trials at reducing pain after tDCS treatment in FM patients. In addition, most trials had a high risk of bias due to inadequate blinding. Although our study did not overcome these shortcomings of non-drug treatment due to the nature of the study, the merits of this study over the previous studies need to be discussed. First, we recruited more participants than the previous studies to get sufficient power for generalization. In addition, because it is impractical to blind the participants in the setting of such trials, we focused on the effect of tDCS in patients who were refractory to conventional treatment as an add-on treatment. In this study, the pain VAS scores after tDCS significantly decreased compared with pre-treatment. Thus, although randomized trials are needed to determine the true efficacy of the treatment, tDCS may be used in FM patients refractory to conventional treatment or in patients who cannot take medications.

The mechanism through which tDCS treatment has an effect remains unclear. Based on functional magnetic resonance imaging (fMRI), an increased level of γ-aminobutyric acid (GABA) in the anterior insula and decreased levels of glutamate and glutamine (Glx) in the anterior cingulate were found in FM patients following tDCS treatment compared with baseline^20^. Moreover, other studies showed that tDCS may affect excitability by modulating the resting membrane potential based on fMRI data during and after stimulation. Based on fMRI data from 12 patients with FM, repetitive M1 tDCS stimulation can change the functional connectivity of regions under the electrode and structurally connected regions such as the thalamus^21,22^. According to this mechanism, it is possible that changes in functional connectivity between the thalamus and brain regions are involved in pain perception. Thus, tDCS may affect the level of neurotransmitters and change the functional connectivity of the stimulated region.

We divided the FM patients into two groups according to pain severity, and patients with more severe pain showed a greater decrease in pain VAS scores than those with less pain after tDCS treatment. Previous studies^23,24^ that have used pain VAS scores as an outcome measure in the study of FM have classified severe pain as a score above 7.5. Because we assessed it in integer units, we chose a cutoff value of 8. Because no study has compared FM patients based on pain severity, it is notable that tDCS appears to have favorable analgesic effects in FM patients and is more effective in patients with severe pain.

We observed a significant pain reduction in tDCS during and after treatment, but the effect of tDCS did not persist to 1 month. In most trials, the effect after tDCS was not explicitly mentioned, and if reported, the duration of the effect was variable. Fregni et al.^6^ showed that the tDCS effect in reducing pain severity did not persist for several weeks after treatment. In addition, Valle et al*.*^7^ showed that tDCS resulted in decreased pain that lasted up to 2 months after the stimulation. The favorable effect of one session of tDCS stimulation was not maintained until the end of the follow-up period in previous trials. On the other hand, we performed booster tDCS treatment in 16 patients. Booster tDCS was effective in all patients, and these patients were not prescribed additional medication or increased doses of conventional treatment during the booster treatment. Based on the above results, booster tDCS may be used for pain exacerbations in FM patients who had initially responded to tDCS.

We also observed a significant improvement after 1 week of tDCS treatment based on FIQ. According to systematic reviews^9^, all tDCS studies in which FIQ was assessed as a measure of functional disability showed transient improvement after follow-up. This finding is consistent with our study, which also showed short-lived improvement in FIQ. Nevertheless, when we compared the slope of pain VAS scores, the trend of the slope of FIQ during follow-up was similar. The improvement in FIQ may be due to the combined effect of pain reduction or patient expectations after tDCS.

We observed a significant improvement in depression after tDCS in FM patients. Of the three trials focusing on tDCS by Fregni et al., Valle et al., and Antal et al., there was no significant change in BDI after M1 stimulation^9^. According to Fregni et al*.*, when the patient brain is stimulated in different locations such as the dorsolateral prefrontal cortex, it can have anti-depressive effects in FM patients^6^. Although we stimulated the M1 cortex of FM patients, we did observe an effect on depression. This may be because depression is a chronic response to pain and decreases in pain may improve depression. Cortical brain areas implicated in depression include the dorsal and medial prefrontal cortex, the dorsal and ventral anterior cingulate cortex, the orbital frontal cortex, and the insula^25^. In addition, pain-related brain regions include the prefrontal cortex, anterior cingulate cortex, insula, amygdala, hypothalamus, periaqueductal grey, rostral ventromedial medulla, and dorsolateral pons/tegmentum^26^. The brain regions involved in depression and pain are closely related. One longitudinal study reported a bidirectional relationship between depression and pain^27^. The intensity of the nociceptive pain stimulus has been associated with activation of central sensitization and psychological factors including depression^28^. Therefore, the antidepressant effect in this study may be due to the sequential effect of improving pain, and not the depressive symptom itself. In addition, M1 cortex stimulation may be one of the targets for the treatment of depression in patients with FM.

Our study showed an improvement in fatigue after tDCS treatment. Fatigue in FM patients is a complex manifestation affected by various factors such as pain intensity, muscular tenderness, psychological distress, and low physical function^29,30^. Thus, the management of fatigue can be challenging for FM patients. Similarly, decreases in fatigue may be considered an additional effect of decreased pain. Therefore, tDCS may decrease fatigue in FM patients.

Lastly, there was no serious adverse event during and after tDCS treatment in this study. Based on a previous systematic review^9^, the most common side-effect of tDCS is a tingling sensation, nausea, headache, and dizziness. A meta-analysis by Hou et al. showed that the most common adverse effects were skin discomfort at the stimulation site, headache, neck pain, and dizziness^11^. A study by Fagerlund et al. showed that 21% of patients had adverse events such as neck pain, scalp pain, itching, burning sensation, sleepiness, trouble concentrating, mood changes, and skin redness^31^. Fregni et al. reported an adverse event in 27% of the M1 stimulation group and 9% of the sham stimulation group, and 1 patient was withdrawn due to headache^6^. Similar to previous reports, we found adverse events in 17.4% of patients and all reported events were minor and well tolerated. Thus, these findings support that tDCS is a safe treatment modality for FM patients.

Our study was performed prospectively and in conjunction with routine clinical practice. The majority of FM trials do not allow concomitant medications and most FM patients are unwilling to discontinue their medications to participate in the clinical trial. Therefore, we assessed tDCS stimulation as an add-on treatment for FM patients with severe pain. In addition, we evaluated various parameters to explore the efficacy of tDCS treatment on pain and associated symptoms. As a result, we found that tDCS may play an important role in improving chronic pain, depression, and fatigue in FM patients. In addition, by showing that tDCS is more effective in patients with severe pain, this study suggests that tDCS could be used in clinical practice as a treatment modality.

There were some limitations in this study. First, the participants were aware of their treatments, and a high expectation of treatment benefit, including a placebo effect, could have influenced the assessment of our results due to a lack of blinding and control groups. Conducting a true double-blind trial remains challenging in the absence of a valid sham comparison group for a non-drug treatment like tDCS. Nonetheless, in an attempt to mitigate the influence of preexisting beliefs and expectations, we explicitly informed potential participants that the study was designed to test the effects of a brain stimulation technique. Although we hoped to decrease expectations and minimize bias by not mentioning tDCS specifically, we recognize the limited generalizability of this trial due to inadequate blinding and limitation of single group study. Second, our study was conducted in a single tertiary care center. Thus, we could not represent characteristics of all FM patients and these results may not be generalizable. Third, we could not determine the optimal number of stimulation sessions, treatment interval, and booster regimen. Longer trials are required to establish the treatment regimen.

## Conclusion

We showed that tDCS has beneficial effects on pain, quality of life, and fatigue in refractory FM patients. Particularly, tDCS is effective at relieving pain severity in patients with severe pain. Our findings support the use of tDCS as a promising treatment in FM patients. Larger, well-designed, unbiased investigations of tDCS treatment are required to explore the mechanism of action, as well as the long-term risks and benefits, and to confirm our results in FM patients.

## Data Availability

Full original protocol and dataset can be accessed upon request for academic researchers by contacting Professor Shin-Seok Lee (shinseok@chonnam.ac.kr).
